# The Promise of Remote Patient Monitoring

**DOI:** 10.1089/tmj.2024.0521

**Published:** 2024-12-06

**Authors:** Bree E. Holtz, Frank A. Urban, Jill Oesterle, Roger Blake, Androni Henry

**Affiliations:** ^1^ Deparment of Advertising and Public Relations, College of Communication Arts & Sciences, Michigan State University, East Lansing, Michigan, USA.; ^2^ Spartan Innovations, East Lansing, Michigan, USA.; ^3^ Michigan Center for Rural Health, East Lansing, Michigan, USA.; ^4^ Merit Network, Ann Arbor, Michigan, USA.; ^5^ Blue Cross Blue Shield of Michigan, Detroit, Michigan, USA.

**Keywords:** remote patient monitoring (RPM), telehealth, wearable devices, artificial intelligence, digital infrastructure, telemedicine

## Abstract

The promise of remote patient monitoring (RPM) lies in its ability to revolutionize health care delivery by enabling continuous, real-time tracking of patient health outside traditional clinical settings. The COVID-19 pandemic accelerated the adoption of RPM, particularly in underserved and rural populations, highlighting both its potential and the persistent barriers that limit its widespread use. This paper explores the critical role of technological advancements—such as wearables, artificial intelligence (AI), and broadband expansion—in sustaining and optimizing RPM in the postpandemic era. We examine Michigan as a microcosm of national health care challenges, focusing on its diverse population and geographic barriers, and propose condition-specific RPM protocols to address these inequities. Key facilitators and barriers to RPM implementation are discussed, with a focus on AI integration, community engagement, and digital infrastructure. We also explore the role of policy reform and public–private partnerships in supporting RPM’s scalability and long-term sustainability. Our findings suggest that while RPM offers a powerful tool for improving health care access and outcomes, especially for chronic conditions and rural maternal health, sustained investment in technology and infrastructure is critical. By addressing these challenges, RPM can become a cornerstone of modern health care, reducing disparities and improving care delivery for underserved populations.

## Introduction

Remote patient monitoring (RPM) holds enormous potential for revolutionizing health care by extending the ability to track patient health beyond traditional clinical settings. The COVID-19 pandemic highlighted the necessity of such solutions, particularly for rural and underserved populations with limited access to health care.^1,2^ As we look toward the future, RPM offers an opportunity to create a more inclusive and accessible health care system, especially as technology advances.

The digital tools for RPM—from wearable devices to home monitoring kits—are already in place, allowing health care professionals to monitor and intervene in real-time.^3,4^ Yet, despite the effectiveness of these technologies during the pandemic, RPM remains significantly underutilized, especially in rural regions that stand to benefit the most. This underutilization stems from several persistent barriers, including insufficient broadband access, limited digital literacy, and the challenges of integrating RPM technology into existing health care systems. Furthermore, health care provider shortages in these areas exacerbate the issue, alongside questions regarding the utility and feasibility of recommending RPM in clinical practice. Concerns about reimbursement policies also contribute to hesitancy, suggesting that RPM must be better supported through infrastructure improvements and policy reforms to realize its full potential as a lasting component of health care delivery. Recognizing RPM’s future potential as a permanent fixture of health care delivery rather than a temporary solution in times of crisis is crucial.

To ensure the long-term success and sustainability of RPM, several critical factors must be considered. This paper explores Michigan’s unique health care challenges, the development of condition-specific RPM protocols, and how advances in technology, infrastructure reform, and community engagement will shape the future of RPM. Through these elements, we aim to highlight the facilitators and barriers that will either enable or impede RPM adoption across diverse populations and geographies.

## Michigan: A Microcosm of U.S. Health Care Challenges

Michigan represents a unique and diverse setting that mirrors many health care challenges faced across the United States (U.S.). With a mix of urban, suburban, and rural areas, Michigan exemplifies the prevalent health care access and quality inequities nationwide.^5^ The state’s rural regions, in particular, face significant barriers to care, such as provider shortages, limited broadband access, and a high prevalence of chronic conditions.^6^ These challenges make Michigan an ideal testbed for implementing and evaluating RPM protocols. Moreover, Michigan’s diverse population, including significant racial, socioeconomic, and geographic variations, allows for a comprehensive evaluation of how RPM can address health care inequities. Lessons learned from the deployment of RPM in Michigan can be applied to similar regions across the U.S., providing valuable insights into how condition-specific RPM protocols can improve health outcomes on a national scale.

## Recommendation for Condition-Specific Remote Patient Monitoring Protocols

We recommend developing and implementing condition-specific RPM protocols to improve health care access and quality in Michigan, particularly for patients with chronic conditions. These protocols would establish standardized guidelines for health care teams, enabling them to remotely monitor and manage chronic diseases more effectively. By integrating wearable devices, home monitoring kits, and telehealth platforms, the RPM protocols would support a comprehensive, patient-centered approach to care. Structured guidelines for remote monitoring and care delivery would enhance clinical decision-making and ensure higher-quality care for patients who face challenges accessing routine, in-person medical services.^7^

Recent studies demonstrate the efficacy of RPM in improving patient outcomes and reducing health care utilization. For instance, many researchers have found a significant reduction in readmissions, emergency room admissions, and mortality rates,^5–6^ highlighting the potential for RPM to improve care quality while lowering costs.^8,9^ These findings underscore the potential for condition-specific RPM protocols to enhance patient care and outcomes in Michigan and nationally.

The development of RPM protocols should be grounded in evidence-based practices and adapted to meet the unique needs of Michigan’s population. Successful implementation requires addressing critical components identified in the literature, such as patient assessment, equipment selection, data collection parameters, alert thresholds, patient education, the identification of provider responsibilities, intervention protocols, communication channels, data management, security, integration with electronic health records, quality assurance, escalation procedures, patient engagement strategies, legal and ethical considerations, and outcome measurement.^10^ By adopting condition-specific RPM protocols, Michigan can significantly improve health care access and quality for patients with chronic conditions. This approach can potentially reduce health care inequities, enhance patient outcomes, and optimize resource utilization across the state. Furthermore, Michigan’s adoption of these protocols can serve as a model for other states, demonstrating how condition-specific RPM can address health care challenges nationwide. We have provided a case study of how RPM can be implemented.

## The Case for RPM in Rural Maternal Health

**Fig. 1.**
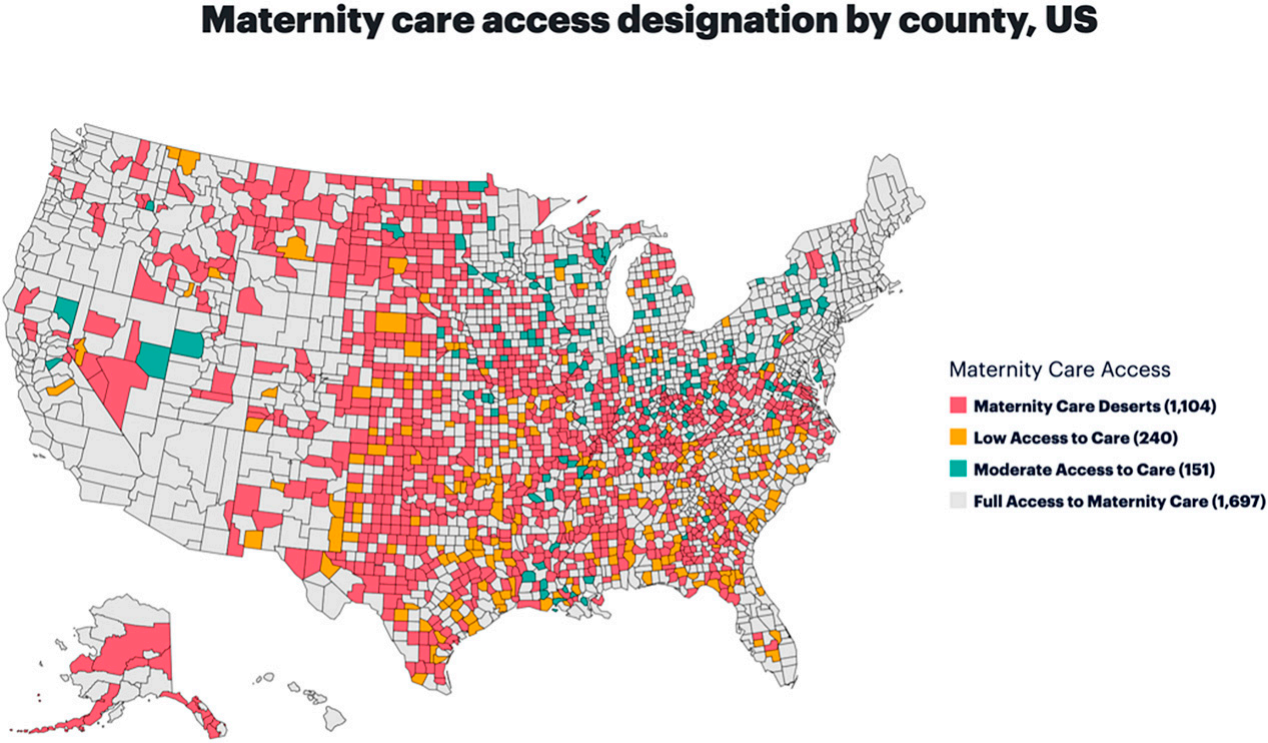
U.S. Maternity care access.

**Fig. 2.**
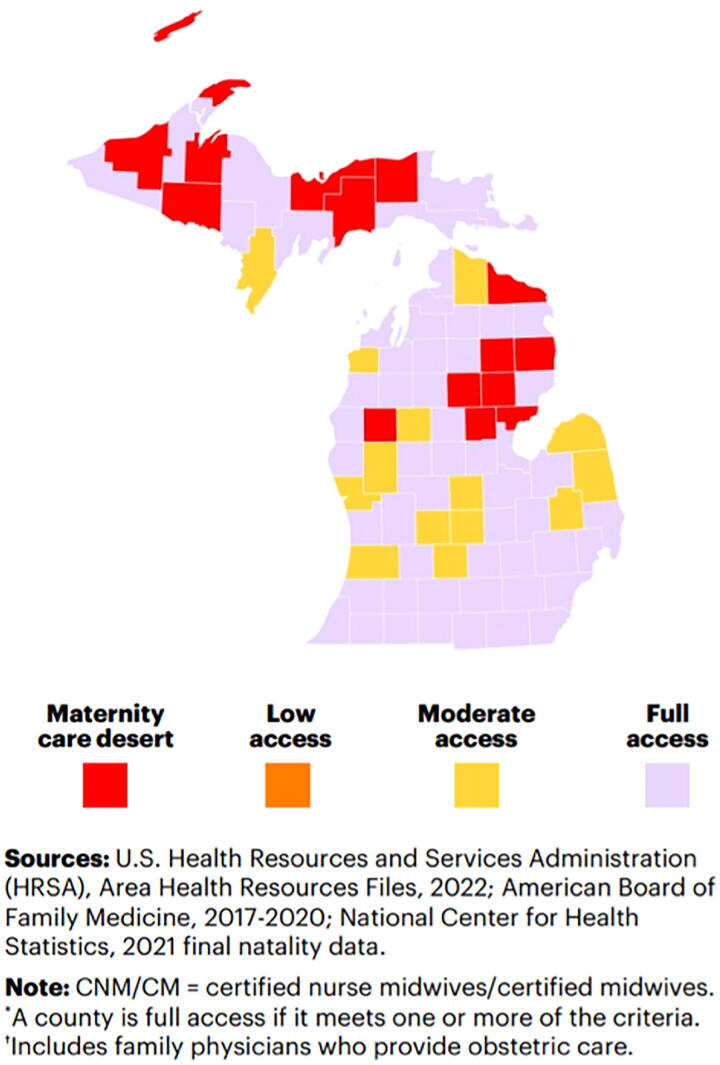
Michigan Maternal Care Access.

Maternal health in rural regions offers a striking example of how RPM can address critical health care gaps. Michigan is one of many states suffering from maternity care deserts, where pregnant persons lack access to essential maternity services. According to a 2022 March of Dimes report, an estimated 6.9 million people and nearly 500,000 births nationwide are affected by limited access to maternity care (see *Fig. 1*). The report identified 13 Michigan counties as maternity care deserts, which means that they lack hospitals providing obstetric care, birth centers, OB-GYNs, and certified nurse midwives. In addition, 17 other counties in Michigan have been classified as having low access to maternity care, with one or fewer hospitals offering OB-GYN services and fewer than 60 OB-GYN providers per 10,000 births (see *Fig. 2*).^11^

With 83 counties in Michigan, the fact that 30 counties fall into either maternity care deserts or low-access zones is alarming. This means that thousands of pregnant persons have limited access to critical prenatal and maternity services. For these individuals, RPM offers a lifeline. By allowing health care providers to remotely monitor key health indicators such as weight, blood pressure, and fetal heart rate, RPM reduces the need for frequent in-person visits and enables timely interventions as the risk of harm increases. This is particularly vital for conditions like preeclampsia, where early detection and management are critical and potentially life-saving. (See Appendix 1)

## The Future Facilitators and Barriers to Implementation

The COVID-19 pandemic highlighted the potential opportunities and challenges of rapidly scaling health care innovations like remote patient monitoring. As we look to the future, several factors will influence the successful implementation of condition-specific RPM protocols, including advances in artificial intelligence (AI), community engagement, and the sustainability and scalability of these initiatives. This section will explore how these elements, shaped by lessons learned during the pandemic, can either facilitate or pose barriers to RPM adoption, ultimately determining its long-term viability in our health care ecosystem.

## AI and Data Analytics

As the landscape of RPM continues to evolve, AI is poised to play an integral role in analyzing the vast amounts of health data generated by remote monitoring devices.^12–14^ The pandemic accelerated the adoption of digital health tools, showcasing the potential of AI to support timely and efficient interventions in health care. AI tools can identify patterns, predict potential health issues, and provide real-time recommendations for interventions, further enhancing the effectiveness of RPM.^15,16^ However, it is essential to approach the integration of AI with caution, addressing concerns related to bias in algorithms, ethical use, transparency in decision-making processes, and the accuracy of AI-generated insights.

The ability to optimize RPM through AI enhances care efficiency and enables more personalized, data-driven health care interventions. By combining RPM with AI analytics, health care providers can offer more precise care, reducing hospital readmissions and improving patient outcomes, especially for those managing chronic conditions.^7^ These advances, driven by lessons learned during the COVID-19 response, are set to propel RPM and AI integration forward, helping health care systems become more resilient and adaptive in the face of future challenges.

## Community Engagement and Education

The COVID-19 pandemic underscored the importance of community engagement and education in adopting digital health tools like RPM. To fully realize the potential of RPM, especially in underserved and rural populations, efforts must prioritize digital literacy, health literacy, and cultural competency. Low digital literacy remains one of the primary barriers to RPM adoption, particularly for vulnerable populations with limited access to technology.^17^ Targeted educational initiatives that teach patients how to use RPM devices and navigate telehealth platforms are essential in bridging this gap.

Health literacy also plays a crucial role.^18^ Without clear, accessible explanations of RPM’s purpose and function, patients may be less engaged with these tools. Providing culturally relevant educational materials can empower patients to take an active role in their care, improving adherence to RPM protocols and health outcomes.

Community health workers (CHWs) can play a vital role in this process by offering hands-on education and support, particularly in areas with limited health care access.^19^ By conducting home visits and assisting patients with RPM setup and usage, CHWs can bridge the digital divide and ensure even those without reliable internet can benefit from RPM. This integration of CHWs into the RPM framework ensures that RPM reaches even the most isolated populations, helping to make health care more equitable.

## Technology Infrastructure

One of the most pressing challenges hindering widespread RPM adoption is the digital divide, particularly in regions like Michigan.^20^ The COVID-19 pandemic brought these inequities into sharp focus, demonstrating that the success of telehealth and RPM hinges on equitable access to technology. In Michigan alone, as of 2023 nearly 500,000 households were unable to be served with high-speed internet access, largely in rural areas where high-speed internet access is critical for telehealth services.^20^

The divide is not just about infrastructure but also about the skills and other resources required to utilize it effectively. In regions where broadband infrastructure is present, families are challenged with adoption because they face affordability, digital literacy, and device access barriers. Approximately 730,000 households in Michigan face significant barriers to connectivity. When combined with those lacking access to necessary infrastructure, nearly 30% of homes across the state are without an affordable, reliable, and high-quality internet connection. Even among those who are connected, many rely on outdated or malfunctioning devices.^20^

RPM and telehealth services become unreliable for these individuals, exacerbating existing health care inequities. Digital literacy also presents a significant challenge, especially among older adults and those in lower socioeconomic brackets.^10^ Patients who lack the skills to use remote monitoring devices or telehealth platforms are often excluded from modern health care solutions, further widening the gap in care access.

## Investing in Infrastructure

Investment in infrastructure and policy reform is essential to address these challenges. Public–private partnerships and state-level investments can help expand broadband access, particularly in rural and low-income areas where health care inequities are most pronounced. Programs that provide subsidized or low-cost internet services and digital devices could dramatically increase participation in RPM, bringing vital health care services to underserved communities.

## Scalability and Adaptability

Pilot programs that target diverse communities can provide critical insights for expanding RPM on a broader scale. These programs allow health care providers to test and refine strategies, ensuring that RPM protocols are adaptable to the specific needs of various populations. Health care systems can optimize RPM’s impact by focusing on scalability and addressing the health care inequities exacerbated during the COVID-19 pandemic.

## Conclusions

The COVID-19 pandemic has demonstrated that RPM and telehealth are temporary fixes and permanent solutions that can significantly enhance the health care system. The technology is here; now, we need the infrastructure and policy backing to realize its full potential. As health care continues to evolve, it is critical to move beyond asking whether RPM works—it does. Instead, we must focus on how to ensure its widespread adoption and integration into everyday health care practices, particularly for the most vulnerable populations.

By investing in digital infrastructure, supporting AI-enhanced RPM solutions, and refining our policies, we can create a health care system that is more resilient, equitable, and responsive to the needs of all communities. Now is the time to act and ensure that RPM remains a crucial tool in the future of health care.

## Appendix 1

### Example Protocol for Remote Patient Monitoring in Rural Maternal Health

To remotely monitor the health of pregnant women in rural areas, particularly those at high risk for complications such as preeclampsia, gestational diabetes, or hypertension, ensuring timely interventions and improving maternal and fetal outcomes.

#### COMPONENTS OF An RPM PROTOCOL

• Patient Identification and Assessment:
  Identify pregnant women in rural areas with limited access to in-person maternity care who are at risk for complications. Initial assessments should include a thorough medical history, current pregnancy status, identification of high-risk factors, and an evaluation of digital literacy and access to technology.
Cultural Competency:
  Ensure that the RPM program is culturally sensitive and addresses the population’s unique needs. This includes providing educational materials in the patient’s preferred language and ensuring that health care providers are trained in culturally competent care, especially in regions with diverse populations.
**Equipment Selection:**
  Provide patients with culturally appropriate, easy-to-use wearable devices and home monitoring kits that allow for the regular collection of vital signs. Equipment may include:
  o Blood pressure monitors
  o Weight scales
  o Fetal heart rate monitors
  o Glucose monitors for those at risk of gestational diabetes

- **Access to the Internet and Technology Support:**
  Recognizing that many rural areas have limited internet access, health care teams should assist patients in connecting with local programs that offer internet subsidies or provide mobile internet devices when necessary. In addition, a technology support service should be available to help patients set up and troubleshoot their devices, ensuring that digital literacy does not become a barrier to care.
- **Patient Education and Digital Literacy:**
  Provide tailored education on using monitoring equipment, ensuring that digital literacy is assessed and supported throughout the program.
  o Patients should be given easy-to-understand instructions and access to a support hotline for assistance.
  o Education should also focus on recognizing critical warning signs, such as symptoms of preeclampsia, and understanding when to seek help.
- **Data Collection Parameters:**
  Set clear guidelines for how often data should be collected. For high-risk pregnancies, daily blood pressure and weight readings, weekly glucose monitoring, and fetal heart rate assessments might be required.
- **Alert Thresholds:**
  Establish specific intervention thresholds for each monitored health indicator. *For example, if a patient’s blood pressure exceeds 140/90 mmHg or if significant weight gain occurs over a short period, an automatic alert is sent to the health care provider.*
- **Provider Responsibilities:**
  Health care providers are responsible for monitoring incoming patient data in real-time, responding to alerts, and initiating timely interventions.
  o Providers should have clear protocols for follow-up care, including virtual consultations or in-person visits if necessary.
- **Role of Community Health Workers (CHWs):**
  Community health workers can play a vital role in bridging the gap between patients and health care providers. CHWs can conduct home visits, assist with device setup, provide education, and help patients navigate challenges related to internet access or cultural barriers.
- **Intervention Protocols:**
  Develop intervention plans based on specific conditions. For example: ***For preeclampsia:*** *If elevated blood pressure or symptoms like blurred vision are detected, immediate steps such as medication adjustments or emergent in-person evaluation should occur.*
- **Data Management and Security:**
  Ensure that all patient data are securely transmitted and stored in compliance with HIPAA and other relevant health care privacy regulations. This includes securely integrating data into electronic health records (EHR) for seamless care continuity.
- **Outcome Measurement and Quality Assurance:**
  Continuously evaluate the effectiveness of the RPM program by tracking key outcomes such as:
  o Number of complications detected early
  o Reduction in emergency room visits
  o Patient satisfaction and engagement
  o Reduced maternal morbidity and mortality
